# mSphere of Influence: Hiring of Underrepresented Minority Assistant Professors in Medical School Basic Science Departments Has a Long Way To Go

**DOI:** 10.1128/mSphere.00599-19

**Published:** 2019-09-18

**Authors:** Michael D. L. Johnson

**Affiliations:** a Department of Immunobiology, BIO5 Institute, and Valley Fever Center for Excellence, University of Arizona, Tucson, Arizona, USA

**Keywords:** assistant, diversity, faculty, fellow, minority, pipeline, pool, postdoctoral, professor, research intensive, underrepresented

## Abstract

Michael D. L. Johnson is a molecular microbiologist observing the role of metals in bacterial biology. In this mSphere of Influence article, he discusses the impact that the paper “Decoupling of the minority PhD talent pool and assistant professor hiring in medical school basic science departments in the US” by Kenneth D. Gibbs, Jr., Jacob Basson, Imam M. Xierali, and David A. Broniatowski (eLife 5:e21393, 2016, https://doi.org/10.7554/eLife.21393) had on him regarding hiring underrepresented minorities at research-intensive institutions and what he considers to be the next steps.

## Abstract (precis)

Michael D. L. Johnson is a molecular microbiologist observing the role of metals in bacterial biology. In this mSphere of Influence article, he discusses the impact that the paper “Decoupling of the minority PhD talent pool and assistant professor hiring in medical school basic science departments in the US” by Kenneth D. Gibbs, Jr., Jacob Basson, Imam M. Xierali, and David A.

## COMMENTARY

As biomedical science departments have grown, a simple question to ask is who has filled those positions? When I look around at other research-intensive institutions, I do not really see a lot of people who look like me peering back. One might assume that it just takes time, that eventually the percentage of underrepresented minorities (URMs; U.S. citizen and permanent resident black, Latinx, and indigenous people) in the population will reflect the number of URM Ph.D.’s, and that that number will be reflected in the number of URM professors. This is why the paper “Decoupling of the minority PhD talent pool and assistant professor hiring in medical school basic science departments in the US” from Gibbs et al. in eLife completely and abruptly changed my viewpoint (1). It is perplexing that there has been no significant increase in the number of URM assistant professor hires per year despite a 930% increase in annual production of (URM) biomedical Ph.D.’s from 1980 to 2013 (1). In fact, with almost 6,000 Ph.D.’s awarded to URMs from 2005 to 2013, there were actually six fewer URM assistant professors in 2014 than in 2005 compared to an 8.6% growth rate with well-represented groups (white, Asian, and international) in that time frame (1). Indeed, there was actually a negative trend in hiring from the URM pool of applicants from 1980 to 2013 (1). Although seemingly preposterous without context, it is not difficult to understand why a model presented by Gibbs et al. predicts that even with the current rate of increase of URM Ph.D.’s, a significant growth in URM professors, sans intervention (i.e., increased hiring practices), will still not be achieved by 2080 (1).

Given the sheer numbers of URM STEM Ph.D.’s entering the field, the problem seems to be at the postdoctoral level. Perhaps URM postdoctoral fellows decide in the midst of this training period that they no longer want to be on the academic track. However, the pool of potential URM candidates has gone up 7.6-fold since the 1980s (1). Furthermore, even accounting for the decline in the number of URM Ph.D.’s who no longer want to pursue a research-intensive faculty position from the time of Ph.D. to current postdoctoral position (which has numbers comparable to well-represented groups), the URM pool is still very much present and accounted for (2). The size of the URM pool is large, but the chosen are few. Even with a leaky pipeline, turning up the pressure should work, but like the analogy, it clearly does not. Perhaps a better analogy is an active transporter which allows for people to become assistant professors and that either URMs are not the preferred substrate or they do not have the correct counter ion to make it through. Accordingly, things that will not fix this problem are more ATP (because we have the energy and drive), more substrate (as a URM’s publications and fellowships are indeed no different from ones obtained by well-represented groups), and/or more of the same transporters.

This information particularly struck me because as a URM assistant professor at a research-intensive institution, I often wonder two things. The first is, given these statistics, by what miracle did I beat the odds to get here? This question can easily lead to, and often does with me and other URM assistant professors, a bout of imposter syndrome. For me, this syndrome is especially true after looking at how my degree in music and less-than-stellar grades in undergraduate science classes stack up against the field. The second thing, and the action item, is what can I do to get other URMs in a similar position as myself? I have been given the advice for as long as I can remember in science that the best thing I can do for future URMs is to be successful. While that might be the best thing for me and my institution (which are good things in their own right), it is not the best thing for the next generation of potential URMs as, given the above data, my or other URMs’ success as a professor will still not bring about more diversity in the professoriate. Further, it puts undue stress and pressure on young URM scientists, as if to say “succeed or in the future your whole ethnic group will never be considered again” (extreme, but accurate).

I see a great number of URM Ph.D.’s who nobly go into positions to support URMs and/or to enhance diversity and inclusion in order to act as that needed counter ion not only for their fellow URM Ph.D.’s to go into the professoriate, but to give them the opportunities and mentoring to go into the career of their choice. They cannot do it alone. A well-known quote goes as follows: “where your treasure is, there your heart will be also.” I would argue that this “treasure” comes in three major forms, money, autonomy, and time. If this problem is truly on the heart of research-intensive institutions, then I implore them to supply the money to buy into the process of hiring more URMs; the autonomy for administrators, deans, and chairs to connect their diversity principles to their hiring practices; and the time to go out and investigate the number of great URM candidates out there who are interested in doing fantastic research. After all, “we are here, we are here, we are here!”
